# Influence of bleached and unbleached bagasse cellulose on the rheology of PVA-based bio-composites

**DOI:** 10.3389/fbioe.2026.1849850

**Published:** 2026-06-05

**Authors:** Hind Zoubeir, Mustapha Lamine, Nouha Lahlou, Mohammed Ouazzani Touhami, Said Aniss

**Affiliations:** Laboratory of Mechanics and High Energy Physics, Faculty of Sciences Aîn-Chock, Hassan II University of Casablanca, Maarif Casablanca, Morocco

**Keywords:** bio-composite, oscillatory rheology, PVA–cellulose, steady shear, sustainability, viscoelasticity

## Abstract

The increasing demand for environmentally friendly food packaging materials has driven the development of bio-based polymers reinforced with natural fibers. Poly (vinyl alcohol) (PVA) is widely studied for its film-forming ability, transparency, biodegradability, and suitability for food-contact applications. Cellulose derived from agricultural by-products, such as sugarcane bagasse, represents an abundant and renewable reinforcement that can enhance the structural and functional properties of polymer-based systems. Few studies have systematically investigated PVA composites reinforced with cellulose extracted from sugarcane bagasse via alkaline treatment, and even fewer have compared bleached and unbleached fibers obtained through a controlled extraction process. Since fiber surface chemistry can influence dispersion, polymer–fiber interactions, and processing behavior, such comparisons remain important. Therefore, this study aims to systematically investigate the influence of bleached and unbleached bagasse-derived cellulose on the rheological behavior of PVA-based suspensions. Cellulose was extracted using alkaline treatment, with or without hydrogen peroxide bleaching, and incorporated into PVA solutions at concentrations of 2 wt% and 4 wt%. Steady shear and oscillatory rheological measurements were performed (plate–plate geometry, 1 mm gap, 25 °C). The incorporation of cellulose induced a transition from near-Newtonian to shear-thinning behavior and increased the elastic contribution, particularly at higher fiber loading. Bleached cellulose tended to promote a more homogeneous dispersion and slightly more stable viscoelastic behavior, although further structural characterization would be required to confirm this observation. These rheological changes are relevant for processing operations such as film casting and coating. Overall, this study provides insight into the role of bagasse-derived cellulose in modifying the rheological behavior of PVA systems and highlights its potential for the development of bio-based materials for packaging applications.

## 1 Introduction

**FIGURE 1:**
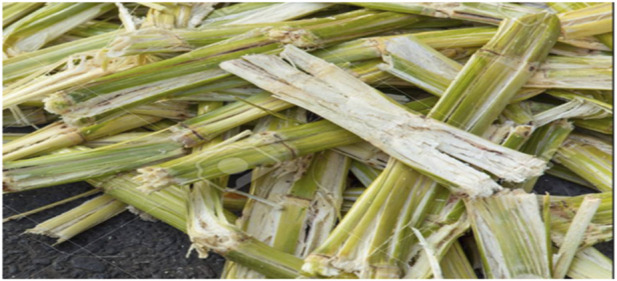
Raw sugarcane bagasse used as cellulose source.

**FIGURE 2:**
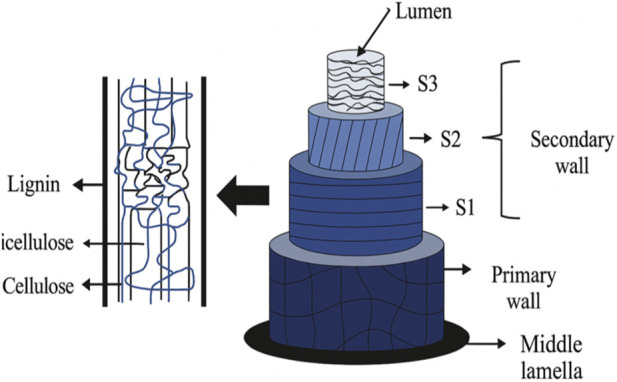
Schematic representation of cellulose fiber structure (Pereira et al., 2015).

The increasing demand for sustainable and environmentally friendly packaging materials has stimulated extensive research on bio-based polymers reinforced with natural fibers. Among these materials, poly (vinyl alcohol) (PVA) has attracted considerable attention due to its film-forming ability, transparency, biodegradability, and compatibility with food-contact applications (El et al., 2017). In this context, the valorization of agro-industrial residues as renewable reinforcement sources has emerged as a promising strategy to improve material performance. Sugarcane bagasse is an abundant and low-cost by-product rich in cellulose (Figure 1), making it an attractive candidate for the development of sustainable polymer composites (Bouftou et al., 2026a). Cellulose derived from bagasse has been incorporated into PVA matrices in various forms, including microcrystalline cellulose and nanocellulose, leading to improvements in mechanical properties, barrier performance, and overall functionality (Diem et al., 2023; Lam et al., 2017; Sarkhel et al., 2023; Dien et al., 2023). These enhancements are generally attributed to improved filler–matrix interactions and the formation of a percolated network within the polymer matrix. Previous studies have shown that the performance of PVA/cellulose systems strongly depends on key factors such as fiber morphology, dispersion quality, and interfacial interactions (Awang et al., 2023; Ahmed et al., 2021; Perumal et al., 2018; Bouftou et al., 2026b). In particular, surface chemistry plays a critical role, as it governs hydrogen bonding and compatibility between the polymer and the reinforcing phase. Advances in cellulose extraction techniques, including alkaline and oxidative treatments, have enabled better control of fiber purity and structure, thereby influencing their reinforcing efficiency (Mamo et al., 2026; El et al., 2015; El Miri et al., 2015). In parallel, increasing attention has been given to the development of bio-based materials for packaging applications, where rheological properties are essential for processing operations such as film casting and coating (Xu et al., 2024; Lamine and Hifdi, 2025; Yaradoddi et al., 2020). The valorization of lignocellulosic biomass requires both efficient extraction strategies and a comprehensive understanding of its structural organization. Environmentally friendly methods have been developed for the isolation of cellulose nanofibrils from sugarcane bagasse, producing high-aspect-ratio nanomaterials with excellent reinforcing capabilities. However, the efficiency of these processes is closely associated with the presence of lignin, a complex aromatic polymer that imparts rigidity to plant cell walls and limits cellulose accessibility, thus requiring controlled modification or removal during processing (Feng et al., 2018; Feofilova and Mysyakina, 2016; Chokshi et al., 2022) (Figure 2). Agro-industrial residues such as cassava bagasse represent abundant and underexploited lignocellulosic resources with significant potential for sustainable material production. Their chemical composition and structural characteristics make them suitable candidates for biotechnological applications, particularly in the development of value-added materials. In this context, polymer matrices such as poly (vinyl alcohol) (PVA) have attracted significant interest due to their biodegradability, film-forming properties, and ability to interact strongly with hydroxyl-rich fillers (John et al., 2009; Niinivaara et al., 2021; Xie et al., 2022). The incorporation of cellulose-based nanomaterials into PVA matrices has been shown to considerably enhance the mechanical, thermal, and barrier properties of the resulting composites. Cellulose nanofibrils derived from sugarcane bagasse act as effective reinforcing agents, improving tensile strength and structural integrity. Furthermore, the functionalization of these systems through the incorporation of bioactive compounds has enabled the development of advanced materials for food preservation applications, such as antimicrobial coatings based on PVA/chitosan systems (Otenda et al., 2022; Bouftou et al., 2024; Boussetta et al., 2023). The viscoelastic behavior of PVA-based suspensions directly affects flow, structural organization, and ultimately the final properties of the resulting films. Despite these advances, few studies have systematically investigated the relationship between cellulose extraction conditions, fiber surface modification, and the rheological behavior of PVA-based systems. In particular, direct comparisons between bleached and unbleached cellulose obtained from a controlled extraction route remain scarce. Therefore, this study aims to systematically investigate the influence of bleached and unbleached bagasse-derived cellulose on the rheological behavior of PVA suspensions. Cellulose was extracted using alkaline treatment, with and without hydrogen peroxide bleaching, and incorporated into PVA solutions at different concentrations. A combination of steady shear and oscillatory rheological measurements was employed to establish structure–property relationships and to better understand the role of fiber surface chemistry on the viscoelastic behavior of the system.

## 2 Materials and methods

### 2.1 Materials

Poly (vinyl alcohol) [PVA, 86.5–89 mol% hydrolyzed, Mw approximately 85,000-124,000 g mol^-1^] was used as the polymer matrix. Sugarcane bagasse was used as the cellulose source. Sodium hydroxide (NaOH), hydrogen peroxide (H_2_O_2_) and all other reagents were of analytical grade and used without further purification. Distilled water was used as the solvent throughout the study.

### 2.2 Cellulose extraction from bagasse

**FIGURE 3:**
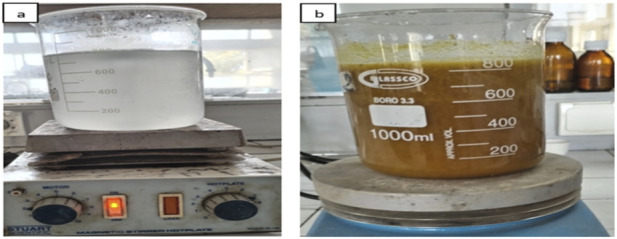
Alkaline treatment of sugarcane bagasse: **(a)** NaOH solution and **(b)** bagasse/NaOH mixture.

**FIGURE 4:**
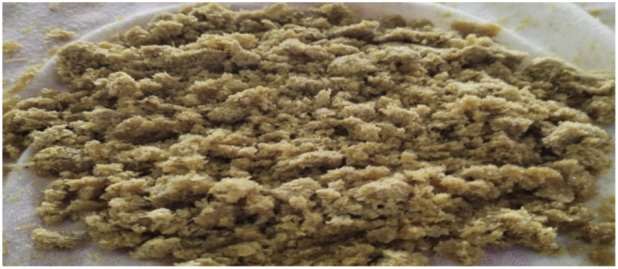
Filtration step after alkaline treatment of sugarcane bagasse.

**FIGURE 5:**
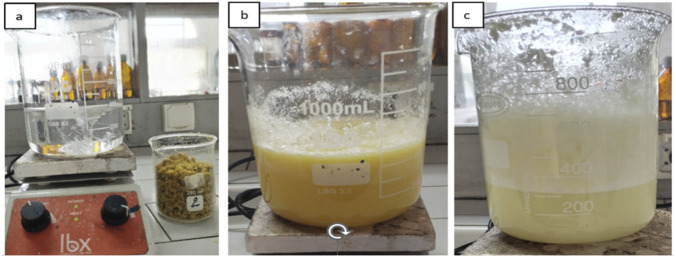
Bleaching treatment of alkali-treated bagasse: **(a)** 4% hydrogen peroxide solution, **(b)** bleaching process, and **(c)** bleaching mixture after 25 min.

**FIGURE 6:**
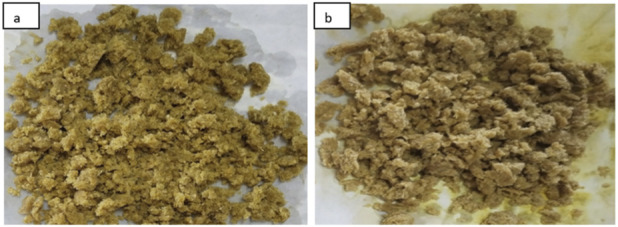
Alkali-treated bagasse fibers without bleaching: **(a)** 2 wt% NaOH treatment and **(b)** 4 wt% NaOH treatment.

**FIGURE 7:**
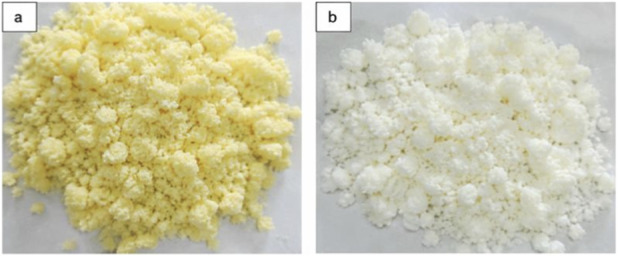
Bleached bagasse fibers after alkaline treatment: **(a)** 2 wt% NaOH followed by bleaching and **(b)** 4 wt% NaOH followed by bleaching.

Sugarcane bagasse was washed, dried and subjected to alkaline treatment in NaOH solution (2 or 4 wt% w/v) at 70 °C for 1 h (Figure 3) under continuous stirring. The treated fibers were washed repeatedly with distilled water until neutral pH was reached (Figure 4). A portion of the alkali-treated fibers was further bleached using 4 wt% (w/v) hydrogen peroxide at 70 °C for 30 min (Figure 5). The unbleached and bleached fibers were oven-dried at 105 °C before incorporation into the PVA matrix. The notation SB is used for unbleached sugarcane bagasse cellulose (Figure 6) and AB for bleached cellulose (Figure 7).

### 2.3 Preparation of PVA–Cellulose suspensions

PVA granules were dissolved in distilled water at 90 °C with mechanical stirring at 500 rpm until complete dissolution. After cooling to room temperature, cellulose fibers were added at 2 and 4 wt% relative to the PVA content. The suspensions were homogenized for 2 min under mechanical stirring and then allowed to rest for 24 h before rheological measurements to promote structural stabilization.

### 2.4 Rheology

Rheology was performed at 25 °C using an Anton Paar MCR 302 using a plate–plate geometry (PP50, 50 mm) with a 1 mm gap. Steady-shear tests spanned 0.1–100 s^-1^. The linear viscoelastic region (LVR) was identified via strain sweep at 1 Hz, frequency sweeps (0.1–100 Hz) were then executed within the LVR to obtain G′ and G″. All measurements were performed at least in duplicate, and the reported results correspond to mean values.

#### 2.4.1 Sample pre-conditioning

Before testing, all suspensions were homogenized (mechanical stirring at 500 rpm for 2 min), pre-sheared at γ̇ = 50 s^-1^ for 60 s and allowed to rest for 300 s to remove flow-history effects. Temperature was maintained at 25.0 °C ± 0.1 °C throughout.

#### 2.4.2 Control of measurement artefacts (wall slip, evaporation, inertia)

To minimize wall slip, sand-blasted PP50 plates (or plates covered with P600 abrasive paper) were used for cellulose-containing suspensions. Gap-dependence tests (0.50, 0.75, and 1.00 mm) were performed, the absence of systematic variation in η(γ̇) was taken as evidence of negligible slip. Evaporation was prevented using a solvent trap and a thin silicone-oil ring around the sample. Oscillatory measurements were restricted to the instrument’s non-inertia-affected window, verified by comparing the measured elastic modulus with the instrument’s inertia baseline.

#### 2.4.3 Determination of the linear viscoelastic region (LVR)

The selected strain amplitude (γ_0_ = 1%) was chosen within the linear viscoelastic region (LVR), as determined from preliminary amplitude sweep tests.

#### 2.4.4 Steady-shear protocol

Steady-shear viscosity was recorded from γ̇ = 0.1 to 100 s^-1^ (10 points per decade, 10 s stabilization per point). Up- and down-ramp curves were compared to assess hysteresis. Constitutive models (Section 3.5) were fitted by non-linear least squares (trust-region algorithm) with 95% confidence intervals for all parameters.

#### 2.4.5 Thixotropy (three-interval test, TTC)

A TTC protocol was conducted: (i) γ̇_1_ = 1 s^-1^ for 120 s, (ii) γ̇_2_ = 50 s^-1^ for 120 s, (iii) return to γ̇_1_ for 600 s. Hysteresis area between up- and down-curves was computed numerically. Rheopexy was only concluded when viscosity increased monotonically and reproducibly during sustained shear; otherwise, the behavior was classified as thixotropic.

### 2.5 FTIR-ATR

The chemical composition of the fibers was analyzed using Fourier Transform Infrared Spectroscopy in Attenuated Total Reflectance mode (FTIR-ATR). This technique enables the identification of the different functional groups present in the material based on their characteristic molecular vibrations. The spectra were recorded over a wavenumber range of 4000 to 500 cm^-1^, which covers the main absorption bands associated with typical chemical bonds in the fibers. A spectral resolution of 4 cm^-1^ was selected as an optimal compromise between spectral precision and acquisition time. To enhance the signal quality and minimize experimental noise, each spectrum represents the average of 32 successive scans.

### 2.6 X-ray diffraction (XRD)

X-ray diffraction (XRD) patterns were recorded using Cu Kα radiation over a 2θ range of 5°–40°, with a step size of 0.02° and a counting time of 1 s per step. The crystallinity index of cellulose samples was calculated using the Segal method according to CI = (I200 − Iam)/I200% × 100%, where I200 is the maximum intensity of the crystalline peak near 22.5° and Iam is the minimum intensity of the amorphous region near 18°.

### 2.7 Scanning electron microscopy (SEM)

Fiber morphology and composite microstructure were examined by scanning electron microscopy (SEM) at an accelerating voltage of 5–10 kV after Au/Pd sputter coating. The SEM observations were used to qualitatively evaluate fiber surface morphology, fibrillation, dispersion, and the presence of agglomerates within the PVA matrix.

### 2.8 Thermogravimetric analysis (TGA/DTG)

Thermogravimetric analysis (TGA/DTG) was performed from 25 °C to 700 °C at a heating rate of 10 °C/min under a nitrogen atmosphere. The onset degradation temperature at 5% weight loss (T5%), the maximum degradation temperature obtained from DTG curves (Tmax), and the residual mass at 700 °C were recorded.

### 2.9 ζ-potential and dispersion stability

ζ-potential measurements were performed on diluted suspensions using electrophoretic light scattering (Zetasizer Nano ZS, Malvern). Cellulose suspensions were prepared at 0.1 wt%, while the PVA solution was prepared at 1.0 wt%. Measurements were carried out at 25 °C ± 1 °C at selected pH values, with a conductivity of approximately 150 μS cm^-1^ and an equilibration time of 120 s. Dispersion stability was further evaluated using the Turbiscan Stability Index (TSI) over 48 h, and the visual appearance of the dispersions was recorded after storage.

## 3 Results and discussion

### 3.1 Fiber chemistry (FTIR)

**FIGURE 8:**
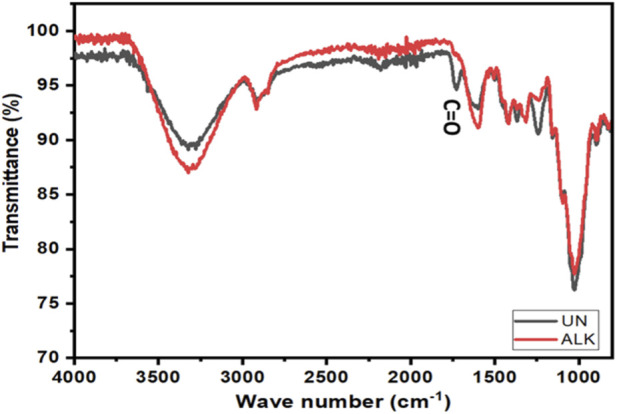
FTIR-ATR spectra of raw, alkali-treated, and bleached bagasse-derived cellulose samples.

FTIR fingerprints (Figure 8) confirm selective removal of hemicellulose/lignin upon alkali treatment and further purification upon bleaching: attenuation near ∼1740 cm^-1^ (C=O groups from hemicellulose esters) and decreased aromatic lignin bands (≈1600–1500 cm^-1^) accompany a relative strengthening of cellulose-associated bands near ∼1030 cm^-1^. These trends indicate higher cellulose purity and improved availability of surface hydroxyls that can hydrogen-bond with PVA. In addition, the broad absorption band observed in the range of 3200–3500 cm^-1^ corresponds to O–H stretching vibrations, characteristic of both PVA and cellulose. This band reflects the presence of hydrogen bonding interactions within the system. Changes in intensity and width may indicate variations in intermolecular interactions between the polymer matrix and cellulose fibers.

### 3.2 X-ray diffraction (XRD)

**FIGURE 9:**
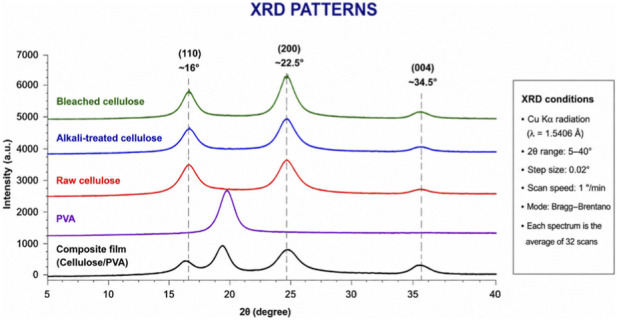
XRD patterns of raw bagasse, treated cellulose fibers, PVA, and PVA/cellulose composite films.

The XRD patterns of raw bagasse, alkali-treated cellulose, bleached cellulose, and PVA/cellulose composite films (Figure 9) revealed noticeable structural changes after chemical treatment. The raw bagasse exhibited a broad diffraction profile with relatively low intensity, indicating the presence of amorphous constituents such as lignin and hemicellulose. After alkaline treatment, the intensity of the characteristic cellulose peak around 2θ ≈ 22° increased, suggesting partial removal of non-cellulosic components and an increase in the relative crystallinity of the cellulose structure. The crystallinity index increased from 61.3% for raw cellulose to 68.7% after alkaline treatment and 73.5% after bleaching, confirming the partial removal of amorphous lignin and hemicellulose fractions. The bleached cellulose showed a sharper and more intense diffraction peak compared with the untreated material, which may indicate improved structural organization and higher cellulose purity after peroxide bleaching. For the PVA/cellulose composite films, the characteristic diffraction peak of PVA near 2θ ≈ 19°–20° remained visible, while cellulose-related diffraction peaks were also observed, indicating the coexistence of both crystalline phases. Slight peak broadening in the composite may suggest intermolecular interactions between PVA chains and cellulose fibers through hydrogen bonding. Overall, the XRD results (Table 1) indicate that alkaline and bleaching treatments improved the structural organization of cellulose and may contribute to enhanced interfacial interactions within the PVA–cellulose system.

**TABLE 1:** Characteristic XRD peaks and crystallinity index of raw cellulose, treated cellulose, PVA, and PVA/cellulose composite films.

Sample	Characteristic peaks (2θ)			Crystallinity index (CI, %)
	Peak 1 (°)	Peak 2 (°)	Peak 3 (°)	
Raw cellulose	15.8	22.3	34.4	61.3
Alkali-treated cellulose	16.1	22.6	34.5	68.7
Bleached cellulose	16.0	22.7	34.6	73.5
PVA	-	19.5	-	-
Composite film (Cellulose/PVA)	19.5	22.5	34.4	-

### 3.3 Scanning electron microscopy (SEM)

**FIGURE 10:**
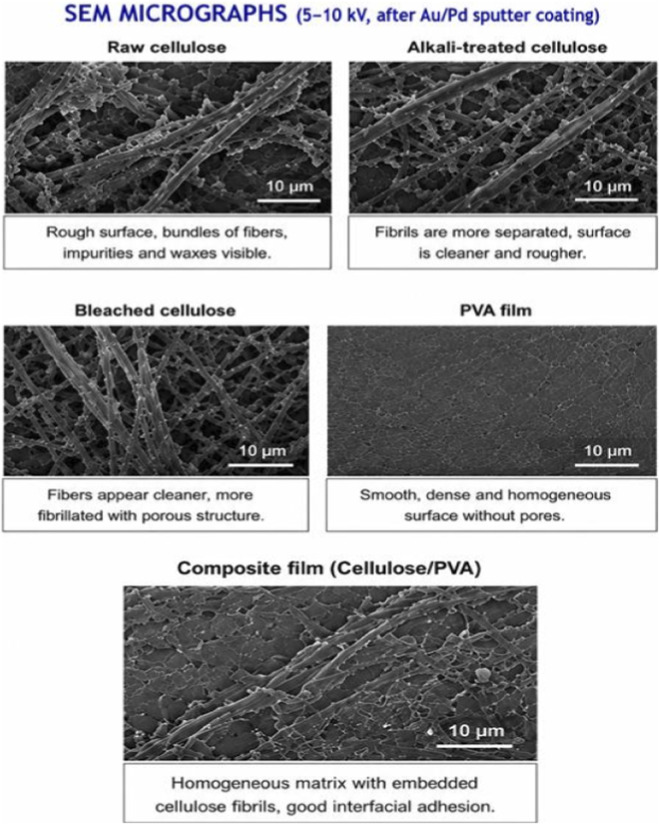
SEM micrographs of raw bagasse, treated cellulose fibers, and PVA/cellulose composite films.

The SEM micrographs (Figure 10) revealed clear morphological changes in the sugarcane bagasse fibers after chemical treatment. The untreated bagasse showed a rough and heterogeneous surface, with compact fiber bundles and visible surface impurities that may be associated with residual lignin, hemicellulose, waxes, and other non-cellulosic components naturally present in the raw biomass. After alkaline treatment, the fiber surface became cleaner and more fibrillated, indicating partial removal of amorphous constituents and disruption of the original fiber bundle structure. The appearance of more individualized fibrils suggests that NaOH treatment promoted fiber defibrillation and increased the accessible surface area of the cellulose. After bleaching, the fibers exhibited a more uniform and purified morphology, with reduced surface impurities and a smoother fibrillar structure, confirming the additional removal of residual non-cellulosic materials. SEM observations of the PVA/cellulose composite films showed that the treated cellulose fibers were relatively well dispersed within the PVA matrix, with no large agglomerates observed at the investigated magnification. This suggests good compatibility between PVA and cellulose, likely supported by hydrogen-bond interactions between hydroxyl groups of both components. Overall, the SEM results indicate that alkaline and bleaching treatments improved the surface morphology of bagasse-derived cellulose and promoted its dispersion within the PVA matrix, which may contribute to the improved rheological stability of the composite suspensions.

### 3.4 Thermogravimetric analysis (TGA/DTG)

**FIGURE 11:**
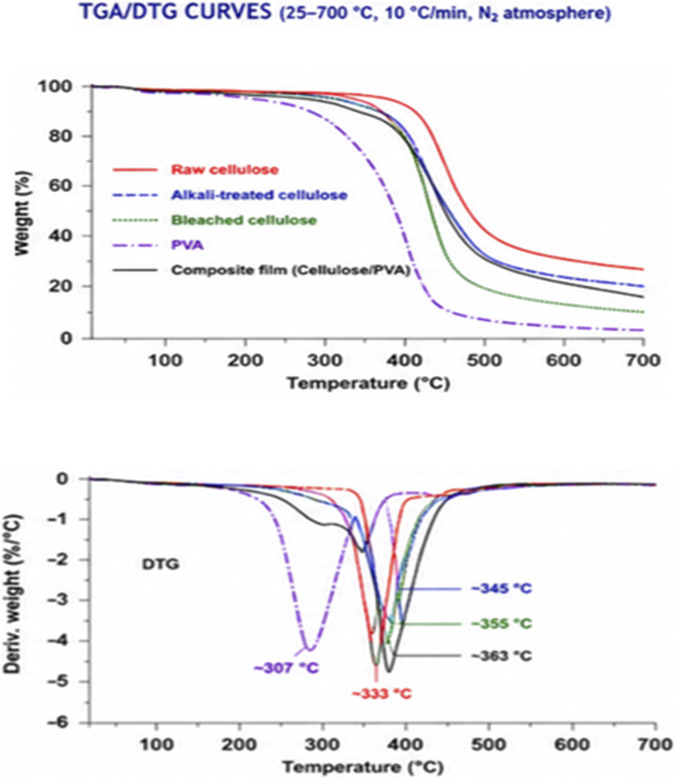
TGA/DTG thermograms of raw bagasse, treated cellulose fibers, PVA, and PVA/cellulose composite films.

The thermal behavior of raw bagasse, treated cellulose fibers, PVA, and PVA/cellulose composites was investigated by TGA/DTG under a nitrogen atmosphere (Figure 11). The thermograms showed an initial weight loss below approximately 100 °C, attributed to the evaporation of physically adsorbed moisture. The main degradation stage occurred between approximately 250 °C and 400 °C and was associated with the decomposition of cellulose and residual hemicellulosic components. As shown in (Table 2), the onset degradation temperature increased from approximately 315 °C for raw cellulose to 325 °C after alkaline treatment and 335 °C after bleaching, indicating improved thermal stability after chemical treatment. This improvement may be attributed to the partial removal of amorphous low-thermal-stability constituents, such as hemicellulose and lignin, and to the increased structural organization of cellulose. The maximum degradation temperature of bleached cellulose was observed at approximately 341 °C, confirming the shift toward improved thermal stability after bleaching. The bleached cellulose also exhibited a more defined degradation profile, suggesting a more homogeneous cellulose-rich structure after purification. Compared with neat PVA, which showed an onset degradation temperature of approximately 250 °C, the PVA/cellulose composite exhibited a higher onset temperature of approximately 320 °C and a Tmax of approximately 363 °C. This suggests that cellulose incorporation improved the thermal resistance of the PVA matrix, probably due to hydrogen-bond interactions and restricted polymer chain mobility. The residual mass at 700 °C was higher for the composite than for neat PVA, which may be attributed to char formation from cellulose and remaining lignocellulosic structures. Overall, the TGA/DTG results indicate that chemical treatment enhanced the thermal stability of bagasse-derived cellulose and that cellulose reinforcement improved the thermal behavior of the PVA-based composite system.

**TABLE 2:** Thermal degradation parameters obtained from TGA/DTG analysis of cellulose samples, PVA, and PVA/cellulose composites.

TGA results (onset temperatures)			
Sample	Tonset/T5% (°C)	Tmax (°C) (Max. Degradation rate)	Residue at 700 °C (%)
Raw cellulose	∼315	∼333	∼12.6
Alkali-treated cellulose	∼325	∼345	∼13.8
Bleached cellulose	∼335	∼341	∼14.6
PVA	∼250	∼307	∼2.1
Composite film (cellulose/PVA)	∼320	∼363	∼8.7

### 3.5 ζ-potential and dispersion stability

**FIGURE 12:**
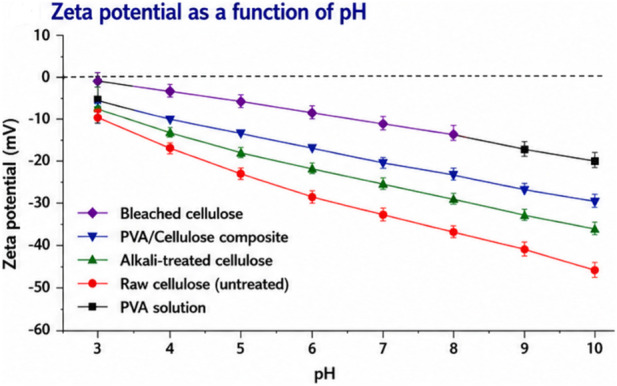
ζ-potential of raw cellulose, alkali-treated cellulose, bleached cellulose, PVA solution, and PVA/cellulose composite suspensions as a function of pH.

**FIGURE 13:**
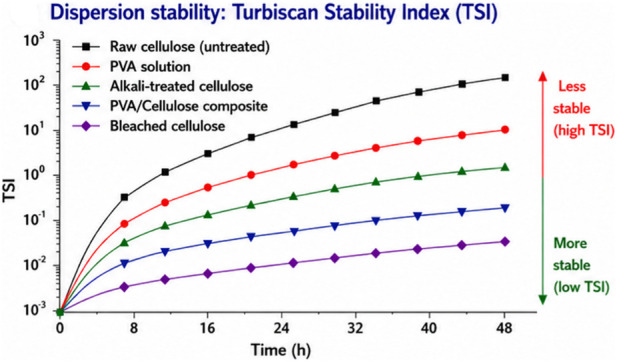
Dispersion stability of cellulose suspensions and PVA/cellulose systems: Turbiscan Stability Index (TSI) evolution over 48 h.

**FIGURE 14:**
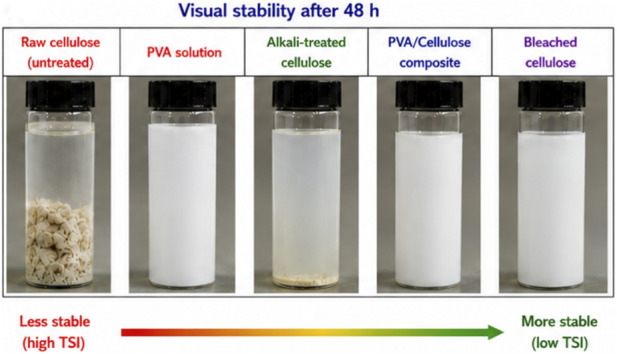
Visual appearance of PVA/cellulose composite films prepared with unbleached and bleached bagasse-derived cellulose.

The ζ-potential and dispersion stability of raw cellulose, alkali-treated cellulose, bleached cellulose, PVA solution, and PVA/cellulose composite suspensions were evaluated as a function of pH and storage time (Figure 12). For all samples, the ζ-potential became progressively more negative with increasing pH, indicating increased ionization of surface hydroxyl groups and stronger electrostatic repulsion under alkaline conditions. At pH 9, raw cellulose exhibited the highest absolute ζ-potential value (−42.3 ± 0.9 mV), followed by alkali-treated cellulose (−35.9 ± 0.8 mV), bleached cellulose (−32.6 ± 0.8 mV), and the PVA/cellulose composite (−31.6 ± 0.7 mV) (Table 3). These values suggest that cellulose-based suspensions reached electrostatic stability at alkaline pH, as absolute ζ-potential values above approximately 30 mV are generally associated with stable colloidal systems. Although raw cellulose showed the highest absolute ζ-potential, the Turbiscan Stability Index (TSI) results revealed poor physical stability during storage, with a strong increase in TSI over 48 h (Figure 13). This indicates that electrostatic repulsion alone was not sufficient to prevent sedimentation or aggregation, probably due to the larger particle size, heterogeneous morphology, and poor dispersion of untreated cellulose. In contrast, bleached cellulose showed the lowest TSI values and the most stable visual appearance after 48 h, indicating improved dispersion stability after chemical purification. The improved stability of bleached cellulose can be attributed to the removal of lignin, hemicellulose, and other impurities, leading to a more homogeneous fibrillar structure and better hydration. The PVA/cellulose composite also exhibited relatively low TSI values, suggesting that PVA contributed to dispersion stability through steric stabilization and hydrogen-bond interactions with cellulose fibers. The visual appearance of the prepared PVA/cellulose suspensions further confirmed the improved homogeneity of the bleached cellulose-based systems (Figure 14). Overall, the combined ζ-potential, TSI, and visual stability results indicate that chemical treatment, especially bleaching, improved the dispersion behavior of bagasse-derived cellulose and enhanced its compatibility with the PVA matrix.

**TABLE 3:** Average ζ-potential values of raw cellulose, alkali-treated cellulose, bleached cellulose, PVA solution, and PVA/cellulose composite suspensions at selected pH values.

Sample	pH 3	pH 5	pH 7	pH 9
Bleached cellulose	−2.9 ± 0.5	−11.0 ± 0.6	−22.3 ± 0.7	−32.6 ± 0.8
PVA/cellulose composite	−5.6 ± 0.4	−13.7 ± 0.6	−22.6 ± 0.6	−31.6 ± 0.7
Alkali-treated cellulose	−6.9 ± 0.5	−15.8 ± 0.6	−25.4 ± 0.6	−35.9 ± 0.8
Raw cellulose (untreated)	−4.3 ± 0.4	−17.2 ± 0.6	−28.7 ± 0.7	−42.3 ± 0.9
PVA solution	−2.2 ± 0.3	−6.6 ± 0.4	−11.2 ± 0.4	−15.0 ± 0.5

### 3.6 Steady-shear flow behavior

**FIGURE 15:**
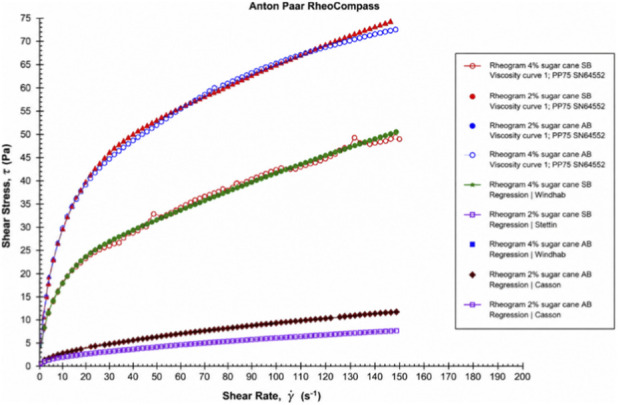
Steady-shear rheograms and model fitting curves of PVA/cellulose suspensions.

**FIGURE 16:**
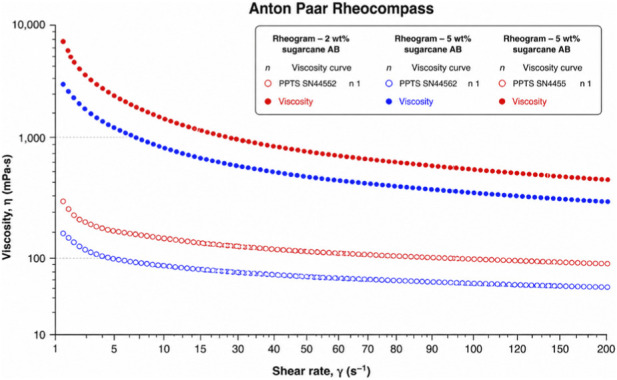
Apparent viscosity as a function of shear rate for PVA/cellulose suspensions.

The neat PVA solution exhibits near-Newtonian behavior (Figure 15), while the addition of cellulose induces a pronounced pseudoplastic response across all formulations (2 wt% and 4 wt%; bleached and unbleached). The viscosity decreases with increasing shear rate (Figure 16), which is advantageous for processing operations requiring low-shear leveling and high-shear spreading. The 4 wt% formulations exhibit higher low-shear viscosity and more stable flow curves, which may be attributed to the formation of a denser internal network resulting from fiber–matrix interactions. Bleached fibers yield smoother rheograms, which may suggest a more homogeneous dispersion.

### 3.7 Time-dependent viscosity

**FIGURE 17:**
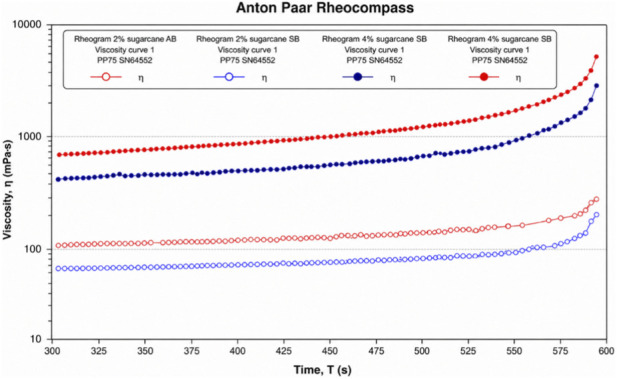
Time-dependent viscosity behavior of PVA/cellulose suspensions under shear.

Under triangular shear-rate protocols and isothermal conditions (25 °C), viscosity tends to increase gradually with time (Figure 17), especially at 4% loading. This slow increase in viscosity may indicate a progressive structuration of the system under shear rather than strict rheopexy.

### 3.8 Small-amplitude oscillatory shear (SAOS)

**FIGURE 18:**
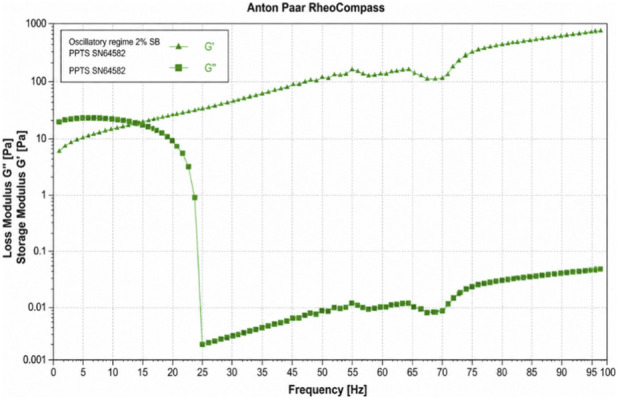
Storage modulus (G′) and loss modulus (G″) of 2 wt% unbleached PVA/cellulose suspension.

**FIGURE 19:**
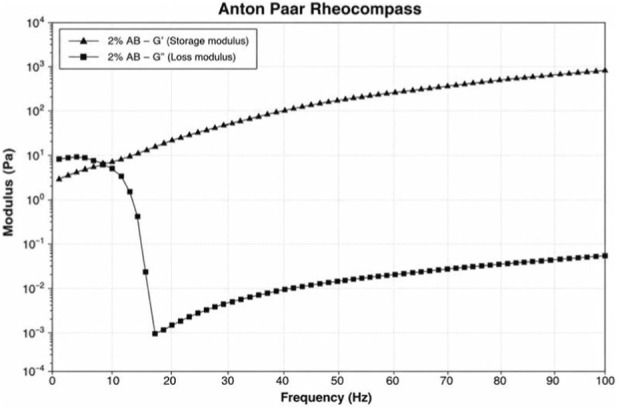
Storage modulus (G′) and loss modulus (G″) of 2 wt% bleached PVA/cellulose suspension.

**FIGURE 20:**
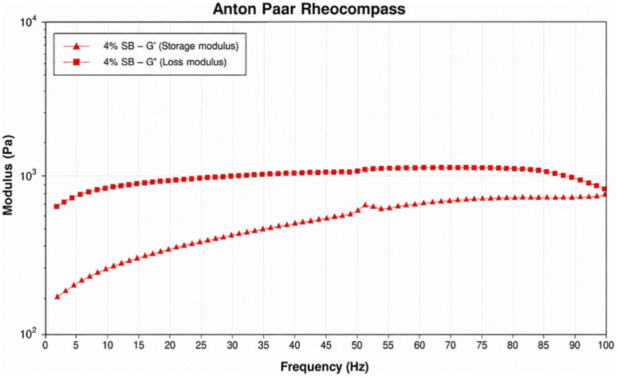
Storage modulus (G′) and loss modulus (G″) of 4 wt% unbleached PVA/cellulose suspension.

**FIGURE 21:**
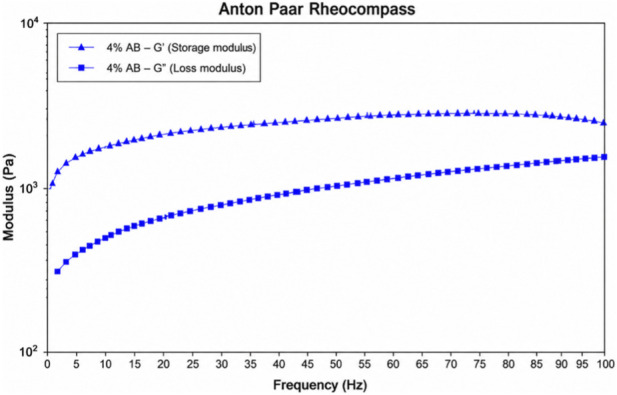
Storage modulus (G′) and loss modulus (G″) of 4 wt% bleached PVA/cellulose suspension.

At low frequencies (Figures 18, 19), all composites display G′ > G″ (elastic-dominant response), more pronounced at 4% loading. The dominance of G′ over G″ at low frequencies indicates the formation of a weak interconnected network within the suspension. Beyond a formulation-specific critical frequency, G′ decreases while G″ increases, indicating progressive structural softening under cyclic loading. At very high frequencies (Figures 20, 21), a slight recovery of G′ may be observed, suggesting short-time elastic resilience. Bleached-fiber suspensions exhibit slightly greater stability across the frequency range, which may be associated with improved interfacial interactions and dispersion homogeneity. This approach allows for a consistent comparison between formulations, as all measurements were performed under identical deformation conditions within the linear viscoelastic regime.

- 2% unbleached curve:
- 2% bleached curve:
- 4% unbleached curve:
- 4% bleached curve:

### 3.9 Rheological model fitting and physical interpretation

The steady-shear viscosity curves were fitted using empirical rheological models selected according to their ability to accurately describe the experimental data. These models are empirical but provide a reliable description of the complex flow behavior of fiber-filled polymer suspensions. In this study, Stettin-type, Windhab-type, and Casson models were found to provide the most suitable fits depending on the formulation. The selection of the appropriate model for each system was based on the goodness-of-fit parameters, including *R* ^2^, RMSE, and AIC values. The Stettin-type model adequately described the flow behavior of low-concentration unbleached suspensions, while the Windhab-type model was more suitable for higher fiber loadings, reflecting increased structural complexity. The Casson model was found to better represent the behavior of bleached cellulose at lower concentrations, suggesting differences in interfacial interactions and dispersion state. Increasing cellulose content (from 2 wt% to 4 wt%) led to a more pronounced non-Newtonian behavior, indicating enhanced structuration of the system. These results highlight the influence of both fiber concentration and surface treatment on the rheological response of PVA–cellulose suspensions. The fitted parameters and corresponding goodness-of-fit metrics are summarized in Tables 4, 5 demonstrating excellent agreement with the experimental data.

**TABLE 4:** Best-fit rheological models for unbleached and bleached PVA/cellulose suspensions.

Formulation	Condition	Representative model	*R* ^2^
2% cellulose	Unbleached (SB)	Stettin-type	0.99
4% cellulose	Unbleached (SB)	Windhab-type	0.98
2% cellulose	Bleached (AB)	Casson	0.97
4% cellulose	Bleached (AB)	Windhab-type	0.99

**TABLE 5:** Strain amplitude applied during oscillatory rheological measurements of PVA/cellulose formulations.

Formulation	γ0 (%)
PVA 5%	1%
2% unbleached	1%
2% bleached	1%
4% unbleached	1%
4% bleached	1%

The transition from near-Newtonian behavior (neat PVA) to pseudoplastic behavior with cellulose loading reflects flow-induced alignment and the break-up and re-formation of weakly bonded fibrillar clusters. Hydrogen bonding between PVA hydroxyl groups and cellulose OH groups may promote the formation of a percolated network that increases low-frequency elasticity (G′) and low-shear viscosity. Increasing the fiber content (from 2 wt% to 4 wt%) further densifies this network, shifting the viscoelastic balance toward elastic dominance at low frequencies while maintaining shear-thinning behavior at high shear rates, which is advantageous for coating processes. Bleaching reduces residual lignin and hemicellulose content and increases the availability of hydroxyl groups, which may enhance interfacial interactions between PVA and cellulose. From a processing perspective, shear-thinning behavior facilitates application by reducing viscosity at high shear, while the increased elastic contribution at low shear improves sag resistance and edge retention. The modest time-dependent structuration observed suggests that controlled rest stages may contribute to stabilizing wet films prior to drying. Future work should focus on establishing quantitative correlations between composition, rheological behavior, and final film properties, as well as on characterizing dispersion (e.g., fibril size and ζ-potential) and evaluating durability under relevant environmental conditions (temperature, humidity, and UV exposure).

## 4 Conclusion

Cellulose extracted from sugarcane bagasse, whether bleached or unbleached, significantly influences the rheological behavior of PVA-based suspensions, leading to shear-thinning behavior and an increased elastic contribution, particularly at higher fiber loadings. The combined steady shear and oscillatory analyses provide insight into how fiber concentration and surface chemistry affect flow behavior and viscoelastic properties. These results suggest that cellulose purity and interfacial interactions play a key role in structuring the polymer matrix and controlling its rheological response under processing conditions. Such behavior is relevant for operations such as film casting and coating, where flow properties and structural stability are critical. Overall, this study contributes to a better understanding of structure–property relationships in PVA–cellulose systems and highlights the potential of bagasse-derived cellulose as a sustainable reinforcement. However, further investigations are required to establish direct correlations between rheological behavior and final material performance in packaging applications.

## Data Availability

The data supporting the findings of this study are available from the corresponding author upon reasonable request.
